# Functional Polymer Composite with Core-Shell Ceramic Filler: II. Rheology, Thermal, Mechanical, and Dielectric Properties

**DOI:** 10.3390/polym13132161

**Published:** 2021-06-30

**Authors:** Andrzej Rybak

**Affiliations:** ABB Corporate Technology Center, Starowislna 13A, 31-038 Krakow, Poland; andrzej.rybak@pl.abb.com; Tel.: +482-222-38488

**Keywords:** core-shell filler, thermal conductivity, epoxy-matrix composite, electrical insulation, dielectric properties, multifunctional composites

## Abstract

Epoxy resin composites filled with ceramic particles are commonly applied in electrification devices as an electrical insulation. In order to maintain proper functionality of such apparatuses it is crucial to optimize a broad range of properties, such as thermal, mechanical and dielectric parameters. In an earlier paper, a novel core-shell filler was developed in order to enhance the thermal conductivity in the epoxy composite used as electrical insulation. The new filler was made of a standard material, which was covered by a thin layer of high thermally conductive shell, namely, alumina coated by aluminum nitride. It was previously shown that the epoxy resin filled with the core-shell Al_2_O_3_@AlN particles showed a significant increase in thermal conductivity with a 63% relative increase. In this paper, a set of complementary measurements was performed and analyzed, namely, rheology, tensile strength, dynamic mechanical analysis, and dilatometry. Moreover, the dielectric permittivity and strength, and electrical resistivity were investigated in order to check if the electrical insulation properties were maintained. The obtained results were compared with the epoxy composite filled with the standard filler. The rheological behavior of the core-shell filled system showed that the processability will not be hindered. The mechanical properties of the composite based on core-shell filler are better than those of the reference system. The coefficient of linear thermal expansion is lower for epoxy filled with core-shell filler, which can lead to better adhesion to internal parts in the electrification devices. The dielectric strength was enhanced by 16% for the core-shell filled epoxy. The investigation clearly demonstrates that the epoxy composite filled with the core-shell particles is an appropriate material for application as electrical insulation with enhanced thermal conductivity.

## 1. Introduction

Current technologies require more and more electricity consumption, therefore the electrification industry, and especially energy distribution technology is still evolving. In order to provide a suitable amount of energy in a safe way, the high effectiveness of the working devices is a key parameter. The proper functionality of the electrical devices can be affected by the several complex factors. The most crucial stresses are the heat, and the dielectric and mechanical factors. Incorrectly selected and electrical insulation materials that are not optimized can lead to the malfunctioning or even damage of the device during its long-term operation in harsh environments [1,2]. One can find extensive research in the literature regarding the investigation of the influence of a harsh environment, such as the temperature and humidity, on the mechanical and long-term properties of epoxy resin-based systems [3,4,5,6].

The materials used as electrical insulation for a medium-voltage electrical apparatus have to combine many parameters, mainly, the dielectric, mechanical, and thermal parameters should be preserved. The correct selection of the polymer matrix, and especially, use of an appropriate filler, allow the manufacture of multifunctional polymer composites for their application in electrical devices [7,8,9,10,11,12,13,14,15].

Epoxy resin is a commonly used material for electrical insulation; it is frequently applied as a potting material or an external cover. Pure epoxy exhibits very low thermal conductivity (0.2 W/m·K) [15], and relatively moderate dielectric and mechanical properties. All these parameters can be enhanced by incorporation of the fillers into the epoxy resin. Alumina (Al_2_O_3_) is often added to epoxy systems in order to encapsulate electrification apparatuses. Due to their high intrinsic thermal conductivity and suitable dielectric properties, alumina particles allow epoxy composites with enhanced properties to be obtained. High filler loading provides a more effective heat transfer through the composite as more heat paths are created [13]. The incorporated filler also provides an efficient load transfer through the composite and as a consequence the mechanical behavior can be improved [16,17]. It is well known that the use of fillers with higher thermal conductivity, such as boron nitride or graphene, can lead to much higher overall thermal conductivity [8,10,12], but their cost is high, and in the case of graphene, which is electrically conductive, the electrical insulation efficiency can be hindered [8,10]. It was shown in our earlier research, that the incorporation of a core-shell type of filler is an appropriate solution, from both a performance and a cost point of view [9,13].

In the previously presented work, it was demonstrated that novel core-shell particles Al_2_O_3_@AlN, which consist of a core made of standard alumina powders and a shell obtained by the formation of an outer thin layer of aluminum nitride (AlN), led to the enhancement of the thermal conductivity of epoxy composite by 63% in relation to the standard Al_2_O_3_ filler, and reaching a value of 2.3 W/m·K [13]. However, in order to assess if a new material can be used as an efficient electrical insulation, several additional parameters should be investigated, such as the processing, dielectric and mechanical properties.

Research works related to epoxy composites filled with different types of core-shell particles can be found in the literature [18,19,20,21,22]. The results indicate that incorporation of the core-shell fillers, such as AlN@Al_2_O_3_ particles or SiC@SiO_2_ nanowires, can increase the storage modulus and improve the thermal conductivity [18,19]. Epoxy composites with multilayer core-shell structured fillers, like Si@SiO_2_@polydopamine and Zn@ZnO@Al_2_O_3_, show an increase in dielectric constant and a reduction in dielectric loss, and the thermal conductivity of the composites is also significantly improved [20,21,22]. Nevertheless, these studies only investigate a limited set of features, and there is not enough emphasis put on the complete analysis of all of the critical parameters, which are highly important from an application point of view.

Therefore, the aim of the presented work was to perform a comprehensive set of measurements in order to assess whether the epoxy system filled with the proposed core-shell particles fulfills the requirements for application. The first part of the research was done in order to confirm that the rheological behavior of the core-shell filled composite is not changed in relation to the reference system. Next, the mechanical, dynamic mechanical, and thermomechanical parameters were examined, and it was found that the overall properties of composite with the modified filler are better than that of the standard system. The last set of measurements indicated that the dielectric behavior of core-shell-based insulation is superior. Thus, the performed investigations proved that the obtained core-shell composite, besides having enhanced thermal conductivity, also exhibits adequate mechanical and dielectric performance and is suitable for application as an electrical insulation material.

## 2. Materials and Methods

### 2.1. Materials Used

#### 2.1.1. Epoxy Matrix

An epoxy (EP) low viscosity hot-curing casting system consisting of an appropriate amount of the following constituents was used for preparation of samples: an epoxy resin, Araldite CY 228-1 derived from Bisphenol A with an epoxy content of 5.0 ± 0.1 equiv/kg; an anhydride hardener, Aradur HY 918; a flexibilizer, DY 045; and an amine accelerator, DY 062. All components were supplied by Huntsman. This resin system is applicable as indoor electrical insulation for a medium electrification device, such as current and voltage transformers [23]. Table 1 shows the properties of the investigated epoxy system.

#### 2.1.2. Standard Filler

Aluminum oxide microparticles ALODUR ZWSK (Treibacher Industrie, Althofen, Austria) were used as a reference filler, hereinafter referred to as STD. Due to its high thermal conductivity and high energy gap, alumina is a commonly used ceramic filler in epoxy composites that are used as electrical insulation. Fused aluminum oxide is a synthetic hard mineral based on alpha-aluminum oxide. It is produced in an electric arc furnace by electro-thermal fusion at temperatures of around 2000 °C. Alumina has a very high hardness, excellent thermal and chemical stability, and a high degree of thermal shock resistance. The basic parameters are presented in Table 2.

#### 2.1.3. Core-Shell Filler

For the investigation, the novel core-shell particles were used, which consist of a core made of standard alumina powders (Al_2_O_3_), and a shell obtained by the formation of a thin outer layer of aluminum nitride (AlN). A detailed description of the process that was used for the synthesis of the core-shell Al_2_O_3_@AlN powders, as well as the in-depth characterization of its physical and chemical structure was presented in our earlier study [13]. The particle size distribution was not affected significantly, whereas a more developed structure of the surface of the Al_2_O_3_@AlN filler was visible—see scanning electron microscopy (SEM) images shown in Figure 4 in our earlier paper [13]. The Al_2_O_3_@AlN core-shell filler is referred to hereinafter as CSF.

### 2.2. Filled Epoxy Samples’ Preparation

Epoxy composites were prepared using the novel core-shell filler and the reference standard filler with a filler content volume (vol.%) of 31, see the list of samples in Table 3. Such filler content is commonly used in the electrical isolation as it is a good compromise between the overall performance of both the uncured liquid mixture used for encapsulation of the device, and the final cured composite.

**Figure 1:**
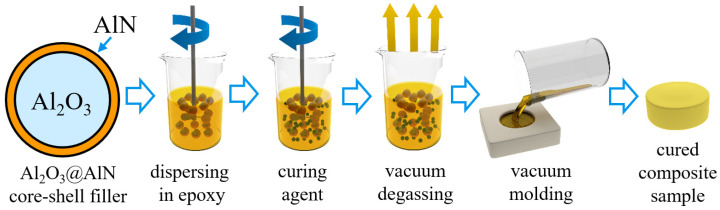
Schematic representation of steps during filled epoxy composite preparation process.

A schematic illustration of the sample preparation steps is shown in Figure 1. First, the filler was dispersed in epoxy and the subsequent constituents, namely, hardener, flexibilizer, and accelerator were added during the mechanical stirring. Liquid composite samples were degassed in a vacuum chamber with up to 2 mbar pressure. Degassing treatment is a crucial step in order to remove air voids, which are introduced during the mechanical mixing. This is a key procedure to avoid a porous structure in the formed composite samples. Manufacturing of samples was performed with the use of a conventional vacuum casting process followed by a curing cycle (4 h at 80 °C and 8 h at 140 °C). Steel molds were used in order to fabricate appropriately shaped samples for measurement. In some cases, waterjet cutting was used to prepare suitably shaped, e.g., dog bone samples for tensile strength tests.

### 2.3. Rheological Measurement

The viscosity measurements were performed by means of Discovery Hybrid Rheometer HR-1 (TA Instruments) with the use of parallel-plates geometry. The initial viscosity as a function of three temperatures (25, 40 and 50 °C) was measured. The shear rate was 10 s^−1^. Measurement was repeated three times to get an average value.

### 2.4. Mechanical Measurements

Tensile properties are the most commonly investigated mechanical properties of filled composites. A Universal Testing Systems 3367 (Instron) was used in order to perform the tensile test according to ISO 527-1 standards [24]. The tensile tests were performed using standardized dog bone-like test samples with eight measurements of each composite type to get significant average values. The test velocity for the Young’s Modulus measurement was set to 1 mm per minute, however, for the determination of the tensile strength test, speed was increased to 5 mm per minute.

Fracture toughness (K_Ic_) was evaluated with the three-point bending method with the use of a single edge notch bend specimen, according to the proper standard [25]. In order to evaluate each parameter, the average value from at least six measurements was taken.

### 2.5. Dynamic Mechanical Analysis Tests

Dynamic mechanical analysis (DMA) was performed by means of DMA 242 E Artemis (Netzsch) in order to evaluate the dynamic-mechanical properties as a function of frequency, temperature and time.

### 2.6. Thermomechanical Measurements

Thermomechanical analysis (TMA) was done by means of TMA 402 F1 Hyperion (Netzsch), and was used for determining the linear thermal expansion, which is an important variable for assessing the dimensional behavior of a material in response to a change in temperature. Specimens in the form of rectangular rods were used.

### 2.7. Dielectric and Electrical Measurements

The relative permittivity and dissipation factor measurements were carried out by means of Solartron 1260 Frequency Response Analyzer coupled with Solartron 1296 Dielectric Interface (AMETEK Scientific Instruments). Flat samples with a 1 mm thickness were used. A system of flat disc electrodes with a guard-ring and a micrometer screw gauge was used [26].

The dielectric breakdown strength was evaluated according to the IEC 60243-1 standard [27]. The breakdown voltage was measured using an unequal steel electrode setup of 25 mm and 75 mm diameter. The measurement setup consisted of a 60 kV test transformer, an electrostatic kilovoltmeter and a voltage regulation system. All of the tested samples were placed in an oil bath in order to limit the surface flashover. Dielectric strength tests were performed at a voltage of AC 50 Hz.

The electrical resistivity of epoxy composites was measured with a Keithley High Resistance Meter 6517B. Cylindrical shaped samples with a diameter of 35 mm and thickness of 1 mm were used. Electrical resistivity measurements were performed at room temperature using a four-point probe at a voltage of 50 V.

All dielectric and electrical tests were performed using standardized test samples with thickness of 1 mm and were carried out 3 times for each composite type to get an average value.

## 3. Results and Discussion

### 3.1. Rheological Parameters

**Figure 2:**
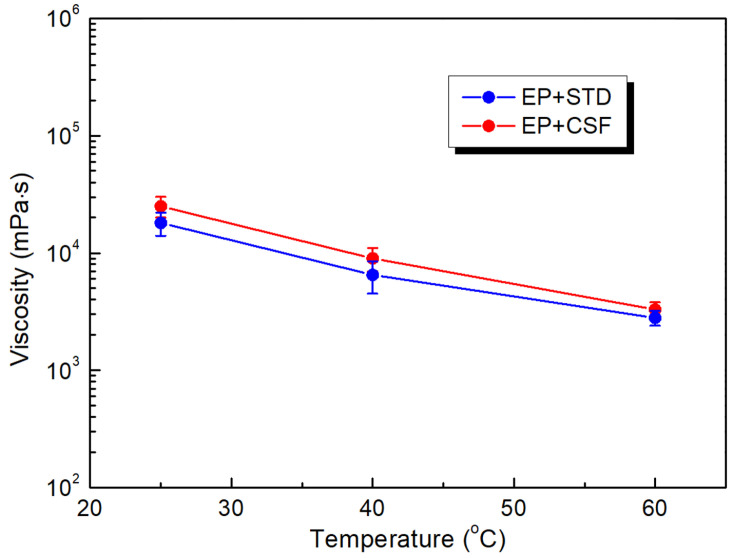
Temperature dependence of the initial viscosity for the epoxy system filled with reference (STD) and core-shell (CSF) particles.

As expected, the viscosity of the liquid epoxy system before curing decreases steadily with an increase in the temperature for both the reference (STD) and the core-shell (CSF) filled samples (see Figure 2). One can see that the viscosity quickly reduced with increased temperature, from 25,021 and 17,852 mPas·s at 25 °C to 3293 and 2812 mPas·s at 60 °C for the EP+CSF and EP+STD composites, respectively. For temperatures higher than 60 °C, the viscosity was not determined, as according to the producer, the beginning of the polymerization process should occur at temperatures above 80 °C and a high increase in viscosity is expected. In the case of a significant increase in the viscosity, one could encounter a problem with the processing of the composite (i.e., filling of the mold cavities, infiltration of windings), and it might hinder effective degassing. Thus, micro-pores can be formed within the bulk of the composite system, which can lead to the faster initiation of partial discharges, and as a result, a decrease in the dielectric breakdown strength. Therefore, the absence of a significant difference in the rheological behavior between both filled systems is a very positive result, as the application of core-shell filler will not hinder the processability of the epoxy system.

### 3.2. Mechanical Properties

Research studies indicate that the mechanical properties of polymer composites can be efficiently improved by incorporation of the ceramic particles into the polymer matrix [28,29]. Furthermore, the mechanical strength strongly depends on the stress transfer throughout the whole polymer composite structure. For composites with particles that are well-bonded to the polymer matrix, the applied loading is transferred between constituents, thus, the overall strength is increased [16,17].

Mechanical tests were performed on the investigated composites in order to check the influence of the core-shell filler addition on the composites’ performance. The obtained results are shown in Table 4. It is worth noting that the mechanical properties, namely, the tensile strength and the fracture toughness of the composite based on the Al_2_O_3_@AlN core-shell filler, were improved in relation to those of the reference system.

As it was shown in previous research work (cf. SEM images of the modified filler structure shown in Figure 4 in [13]), the surface of the Al_2_O_3_@AlN filler is well-developed with more roughness in comparison to the standard filler. Therefore, the mechanical interlocking at the interface of the core-shell filler and epoxy matrix improves the adhesion, and as a result, leads to the improvement of the composite mechanical properties. Especially, the enhancement of the tensile strength and fracture toughness of the composite is clearly visible as shown in Table 4.

### 3.3. Dynamic Mechanical Analysis Results

**Figure 3:**
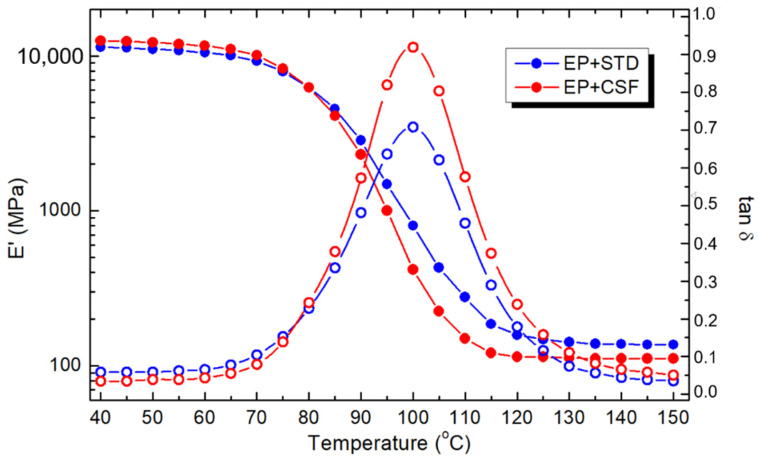
Temperature dependence of the DMA curves of storage modulus *E′* and tan δ for the epoxy system filled with reference (EP+STD) and core-shell (EP+CSF) particles. Measurement done at 1 Hz.

The DMA results for EP+STD and EP+CSF samples are shown in the bottom part of Table 4 and in Figure 3, and they illustrate the α-relaxation associated with the glass transition of the epoxy system. In general, the storage modulus (*E′*) denotes the stiffness of the material, while the loss modulus (*E″*) refers to the amount of oscillation energy transformed into heat. The parameter tan δ reflects the mechanical damping or internal friction of a visco-elastic system.

A small decrease in the glass transition temperature can be noticed, with a slight drop in the tan δ peak temperature and the loss modulus peak temperature. It can be assumed that the temperature at the peak measured from tan δ corresponds to the glass transition temperature *T_g_*. It is worth noting that the unrelaxed storage modulus (*E′*), which was measured at the start of heating at 40 °C, is higher for the EP+CSF sample, as was observed in the case of the modulus from the tensile measurement.

### 3.4. Thermomechanical Behavior

During heating-cooling cycles, the materials undergo changes in their thermomechanical properties. In the case of the thermal expansion of EP+STD and EP+CSF samples, which is shown in Table 5, one can observe that the coefficients of linear thermal expansion are almost identical above the glass transition temperature *T_g_* (which was evaluated by means of DMA), but it is somewhat lower for EP+CSF below *T_g_*. The decrease in the coefficient in the glassy state, along with the reduction below *T_g_*, is beneficial because it can decrease the formation of internal stresses in the cool-down step after curing. As a result, the dimension mismatch to the internal metal parts encapsulated during the casting of electrical devices is hindered [30].

A slight decrease in thermal expansion in the case of EP+CSF can be explained by a lower thermal expansion of the AlN shell in relation to the Al_2_O_3_ core. A lower coefficient of thermal expansion for epoxy filled with core-shell filler, can also lead to the hinderance of delamination at the copper conductor and epoxy interface, what is crucial with regard to the use of epoxy systems in electrical applications.

### 3.5. Dielectric and Electrical Properties

One can also find approaches to control the dielectric properties of the epoxy-based composites by means of the addition of appropriate fillers [31,32]. As mentioned in our earlier study [13], the main aim of the fabrication of the core-shell filler was to modify the thermal conductivity of the epoxy-based electrical insulation system. Therefore, ceramic particles were selected as the electrical insulating material [15] in order not to influence the electrical insulation properties of the composite. To evaluate whether the proposed core-shell filled composite is suitable for application as electrical insulation, the dielectric and electrical tests were performed; the results are presented in Table 6.

One can see that the dielectric constant and dissipation factor measured for the composite filled with the core-shell Al_2_O_3_@AlN particles did not change significantly in comparison to the reference sample filled with alumina. The dielectric constant of the filled composite was not affected, as Al_2_O_3_@AlN and AlN have the same dielectric permittivity value. Especially interesting, from the application point of view in medium-voltage devices, is the observed enhancement of the dielectric strength value from 38 kV/mm for EP+STD to 44 kV/mm (an almost 16% increase) for EP+CSF. This can be explained by the fact that AlN in the solid form has a much higher dielectric breakdown strength, therefore EP+CSF also exhibits a higher dielectric performance. The obtained dielectric and electrical results clearly show that incorporation of the modified filler allows fabrication of an electrical insulation material with properties that are superior to the reference system.

## 4. Conclusions

The developed particles, in the form of a core-shell structure, are very promising fillers that could effectively replace the standard fillers in electrical insulation based on epoxy resin composites. It was shown that despite the enhanced thermal conductivity, which was demonstrated in our previous study, the core-shell filled epoxy composite also possesses other crucial parameters, which are required from an application point of view, namely:

- The rheological behavior of the core-shell filled systems shows that application of the core-shell filler will not hinder the processability of the epoxy system during fabrication of the electrical devices.
- The mechanical properties, namely, the tensile strength and the fracture toughness of the composite based on Al_2_O_3_@AlN core-shell filler, are better than that of the reference composite.
- The dynamic mechanical analysis results indicate that the modified filler has a negligible influence on the glass transition of the core-shell filled composite.
- The lower coefficient of linear thermal expansion measured for epoxy filled with core-shell filler can lead to the hinderance of delamination at the interface of the copper conductor and the epoxy electrical insulation.
- One of the most interesting results is the observed enhancement of the dielectric strength value from 38 kV/mm for the standard system to 44 kV/mm (almost 16% increase) for the core-shell filled epoxy.

The observed enhancement of the thermal conductivity (shown previously), the currently presented superior mechanical and dielectric properties, and the lack of visible influence on the thermal and processing parameters, make the investigated composite with incorporated Al_2_O_3_@AlN core-shell filler an ideal candidate for the manufacturing of electrification devices with either much higher ratings or a reduction in the footprint of electrical apparatuses.

## Figures and Tables

**Table 1:** Properties of the thermosetting resin used for fabrication of filled composites.

Parameter ^1^	Araldite CY 228-1	Aradur HY 918	Flexibilizer DY 045	Accelerator DY 062
Content (pbw)	100	85	20	1.5
Viscosity (mPa·s)	4300	65	122	10
Density (g/cm^3^)	1.15	1.21	1.12	0.9
Flash point (°C)	160	159	235	59
Vapor pressure (Pa)	2·10^−2^	1	<10	300

^1^ Values determined at 25 °C.

**Table 2:** Basic parameters of the reference alumina filler.

Parameter	Value
Density (g/cm^3^)	3.9
*d_50_* (μm) ^1^	6.5
Chemical analysis (%)	Al_2_O_3_ ≈ 99.87; Fe_2_O_3_ ≤ 0.01; Na_2_O ≤ 0.10; SiO_2_ ≤ 0.01
Coefficient of linear thermal expansion (10^−6^ K^−1^)	8
Thermal conductivity (W/m·K)	36
Dielectric permittivity, ε_r_	8.6
Electrical resistivity (Ω·cm)	>10^14^

^1^ Particle size distribution of raw material Al_2_O_3_ is shown in [13].

**Table 3:** Details and definition of the investigated filled epoxy composites.

Sample Name	Filler Type	Filler Content (vol.%)
EP+STD	Al_2_O_3_	31
EP+CSF	Al_2_O_3_@AlN	31

**Table 4:** Mechanical parameters measured for the reference sample and the composite filled with Al_2_O_3_@AlN core-shell particles.

Parameter	EP+STD	EP+CSF
Tensile strength (MPa)	52.87 ± 2.76	57.15 ± 3.59
*E* from tensile test (MPa)	10231 ± 137	11431 ± 219
Fracture toughness *K_Ic_* (MPa·m^1/2^)	2.44 ± 0.21	2.68 ± 0.17
*E′* at 40 °C (MPa)	11361 ± 205	12520 ± 173
*E′_r_* (tan δ peak, *T* + 50 °C; MPa)	135 ± 8	110 ± 6
Tan δ peak *T* (°C)	99.6 ± 0.4	99.1 ± 0.5
Tan δ peak height	0.704 ± 0.005	0.924 ± 0.006
Tan δ FWHM (°C)	27.8 ± 0.2	25.7 ± 0.6
*E″* peak *T* (°C)	84.1 ± 0.4	82.3 ± 0.9
*E″* peak (MPa)	1495 ± 73	1873 ± 18

**Table 5:** Coefficient of linear thermal expansion below and above the *T_g_*.

Coefficient of Linear Thermal Expansion	EP+STD	EP+CSF
< *T_g_* (10^−6^ K^−1^) ^1^	56 ± 4	49 ± 3
> *T_g_* (10^−6^ K^−1^) ^2^	108 ± 6	106 ± 4

^1^ Average value evaluated for temperature range from 30 °C to 70 °C. ^2^ Average value evaluated for temperature range from 120 °C to 140 °C.

**Table 6:** Dielectric parameters and electrical resistance obtained for the reference system and the composite with the core-shell particles.

Parameter ^1^	EP+STD	EP+CSF
Dielectric permittivity, ε_r_	4.3 ± 0.2	4.1 ± 0.3
Dielectric dissipation factor, tan δ (× 10^−3^)	4.6 ± 0.2	4.4 ± 0.1
Breakdown strength (kV/mm)	38 ± 3	44 ± 2
Electrical resistivity (Ω·cm)	>10^14^	>10^14^

^1^ Values measured at 25 °C for 50 Hz.

## Data Availability

Data are available on request.
